# Hydrophobic surface modification and characterization of melamine foam

**DOI:** 10.55730/1300-0527.3563

**Published:** 2023-04-17

**Authors:** Merve OKUTAN, Filiz BORAN, Ayça ERGÜN, Yusuf KANCA, Bengi ÖZKAHRAMAN, Hüseyin DELİGÖZ

**Affiliations:** 1 Department of Chemical Engineering, Hitit University, Çorum, Turkey; 2 Department of Chemical Engineering, İstanbul University-Cerrahpaşa, İstanbul, Turkey; 3 Department of Mechanical Engineering, Hitit University, Çorum, Turkey; 4 Department of Polymer Engineering, Hitit University, Çorum, Turkey

**Keywords:** Melamine foam, oil-water separation, LbL, dip coating, silica

## Abstract

Superhydrophobic and oleophilic modification of commercial acoustic melamine foam (MF) was made in this study. The modification was carried out with chitosan (CHI) and silica particles (SiO_2_), by using both a layer-by-layer-like approach (LbL) and dip coating technique. Subsequently, 1-octadecanethiol was used as a secondary modification agent. QCM-D, SEM, and FTIR analyses confirmed that the coating was successfully performed. After the modification, the column wall thicknesses increased than that of MF and they ranged from 25% to 48% for modified MF with an LbL-like approach (MMF) and modified MF via dip coating technique (MMFd), respectively. The sorption experiments showed that modified MFs, which had a water contact angle (WCA) above 160°, could sorb several model pollutants (vegetable oil, chloroform, ethanol, and toluene) up to 76–130 times their original weight. It had been determined that MMF protects its open-pore structure better than that of MMFd, which indicated that MF has a more uniform pore structure after modification. Furthermore, after 10 cycles of the sorption and release process, there was no significant change in sorption capacity, and they preserved their mechanical stability and flexibility.

## 1. Introduction

Polymer foams have unique chemical and physical properties owing to many tiny interconnected holes inside porous polymeric structures. Compared with bulk structured polymeric materials, polymer foams show overwhelming advantages, such as low density, lightweight, eco-friendly process, high impact strength, and excellent thermal/acoustic insulation. Polymeric foams are convenient for an extensive variety of industrial applications for instance construction, transportation, energy storage devices, oil-water separation, and water treatment [1,2]. Moreover, modified foams with new and different properties, which can modify using various techniques and components, have alternative application areas. One of the most attractive approaches for the use of modified foams is superhydrophobic/superoleophilic applications involving nonover wetting conditions. When the surface contact angle is bigger than 150°, it is referred to as superhydrophobic [3]. Based on the literature, the fabrication of hydrophobic polymer foam may be synthesized using different approaches and modification agents depending on the polymer foam characteristics. Preparation of the polymeric foams with low surface tension by deposition of micro- and nanoparticles on the substrate and their further modification with secondary chemicals is an interesting subject. There are many studies on the application areas of these materials based on their superhydrophobic properties by examining the interfacial interactions of these materials with water and oil/organic solvent [4,5]. Among the micro/nanoparticles, SiO_2_ is a commonly used and commercially accessible material due to its superior properties such as good mechanical and thermal properties. Researchers emphasized that silica nanoparticles can change the wettability in porous media. In addition, it was reported to be an advantageous material for converting hydrophilic behaviors to stable hydrophobic ones with fast and easy modifications [4,6,7]. For example, Yang et al. developed hydrophobic thermoset polyurethane (PUR) surfaces decorated with silica nanoparticles to be performed for moisture-proof, self-cleaning, and waterproof materials. They found that the 4% silica nanoparticle doped into the polyurethane increased the contact angle above 150° [6]. Gu et al. reported that SiO_2_ grafted PUR superhydrophobic porous membrane (WCA changed between 152.7° and 154.9°) showed high oil flux and oil absorption capacity, which was used for oil-water separation and oil removal [8]. As seen from the literature, the foam modification was carried out by immersion of the foam directly into the solution containing modification agents such as silica [9], magnetic poly(vinylidene fluoride-co-hexafluoropropylene) [10], polyvinyl alcohol and polydimethylsiloxane [11], magnetic chitosan (CHI) and sodium perfluorononanoat [12], graphene [13], oleic acid-capped TiO_2_ nanoparticles [14], polydivinylbenzene and polydimethylsiloxane [15], polydopamine and silver nanoparticle [16], etc. On the other hand, with a LbL technique, it will be possible to modify the foam surface through bonding or interactions with functionalized inorganic particles without any extra material acting as a binder between the foam and inorganic particles [17]. Furthermore, the amount of organic solvent for polymers and particles can be reduced since most of the polyelectrolytes used for LbL have polar functional groups and some inorganic particles can give stable dispersions at different pH values. There are a few studies on the preparation of hydrophobic and oleophilic foams using the LbL technique with organic and different inorganic materials such as SiO_2_ [17], titanate nanotubes [18], montmorillonite [19] and graphite oxide [20]. Two of these studies dealt with the wettability of the prepared materials by determining the water contact angles. The contact angle of the LbL cellulosic membrane prepared with silica additive was improved from 133° to 151°, while the contact angle of the polyurethane sponge prepared with montmorillonite additive was enhanced from 101.18° to 120.12° [17,19]. Furthermore, in these studies, controlling the roughness by the number of bilayers, preparing with a simple technique, and optimizing the flame-retardant or mechanical properties were reported as advantages.

As far as we know from the literature, there is no study on the modification of melamine foam (MF) with a LbL-like approach with silica particles and the comparison of this technique with direct dipping in terms of physicochemical and mechanical properties. In this study, two routes were followed for the hydrophobic modification of commercial MF. The first one was the modification of MF with silica particles dispersed in the CHI solution used as the adhesive agent by the dip coating technique. The second one was the sequential deposition of CHI and amine functionalized silica particles on the MF in a single layer in the presence of poly(sodium 4-styrenesulfonate) (PSS), which will provide the opposite charge needed for the LbL procedure. As a secondary modification, the foams were subsequently reacted with a hydrocarbon-thiol compound. Quartz crystal microbalance dissipation (QCM-D), Fourier transform infrared spectroscopy (FTIR), scanning electron microscopy (SEM) and X-ray diffraction (XRD) analyses were made for SiO_2_ particles. Also, the surface and morphological and structural properties of the foams were analyzed using SEM and FTIR. Furthermore, the changes in water contact angle (WCA), density, and sorption capacity of foams were studied in detail as well as their mechanical properties using a compressive mechanical test.

## 2. Materials and methods

### 2.1. Materials

Melamine foam (MF, Basotect) from Adetaş; ethanol (CH_3_CH_2_OH, anhydrous) from Carlo Erba; (3-Aminopropyl) triethoxysilane (APTES, 99%), acetic acid (C_2_H_4_O_2_, glacial, ≥ 99%), tetraethyl orthosilicate (TEOS, ≥ 99%), ammonium hydroxide (NH_3_, 26%), poly(sodium 4-styrenesulfonate) (PSS, Mw: 70,000) and chitosan (CHI, low molecular weight, deacetylated chitin) from Sigma-Aldrich; 1-octadecanethiol (ODT, for synthesis) from Merck were provided.

### 2.2. Synthesis and modification of silica particles

**Figure 1:**
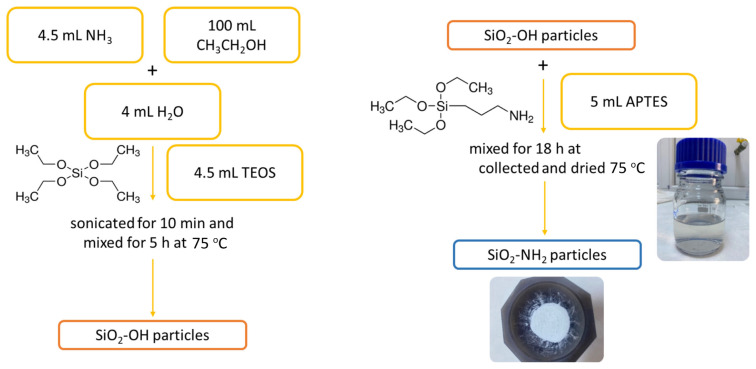
Schematically representation of the silica particle synthesis.

NH_3_ (4.5 mL), water (4 mL), and ethanol (100 mL) were mixed for 10 min and TEOS (4.5 mL) was added drop by drop to this mixture. Then it was dispensed in a probe-type sonicator (probe: TS113, Ampl: 10%, pulse: 0.5s/1s) for 10 min. It was stirred at 75 °C for 5 h. Mixing was continued overnight (approximately 18 h) by adding 5 mL of APTES for amine modification [17]. The resulting mixture was kept in a laminar flow cabinet for 2 h, and then in an oven at 100 °C overnight. For comparison, SiO_2_ nanoparticles were synthesized by applying the same procedures without the addition of APTES (Figure 1).

### 2.3. Modification of MFs

**Figure 2:**
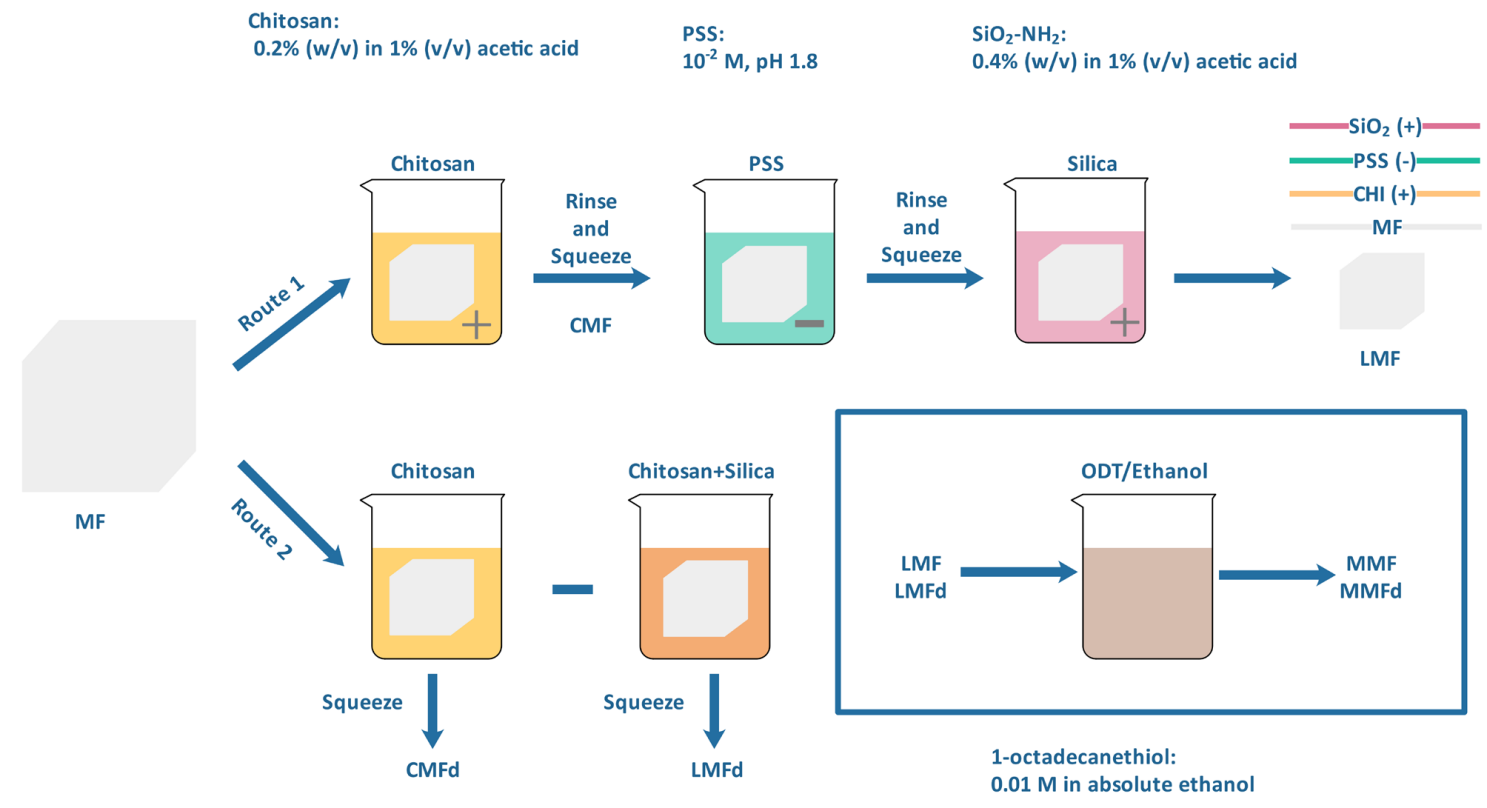
Schematically representation of the foam modification steps and the sample codes.

The modification procedure of the MFs and the applied steps are given in Figure 2 and the foam samples are labeled in Table. MF prewashed with an ethanol-water mixture was modified by using two different routes. In the first, MF was immersed in a 0.2% (w/v) CHI solution (1% (v/v) acetic acid) in which aminated silica particles (0.4% (w/v)) were dispersed. After MF was removed from the solution, the sample (named as LMFd) was squeezed, dried at 70 °C, and stored for further use. In the second, a single-layer coating was made with a LbL-like approach. For this purpose, 0.2% (w/v) CHI (1% (v/v) acetic acid, pH 3.5) and 0.4% (w/v) aminated silica (1% (v/v) acetic acid) solutions were used as the positively charged coating solution while PSS (10^−2^ M, pH 1.8) was used as the negatively charged coating solution. MF was successively immersed (5 min) into positively charged CHI, negatively charged PSS, and positively charged silica solutions. After each dipping step, washing with water (2 min) was performed. Finally, before further use, LbL-like modified MF (LMF) was dried at 70 °C.

An additional modification process was used to increase the hydrophobicity of both LMFd and LMF samples. For this purpose, these final samples were modified with ODT in anhydrous ethanol for 12 h at room conditions. Samples taken in the reaction medium were dried overnight. In addition, CMFd (prepared with direct coating) and CMF (prepared with the LbL-like approach), which are intermediate products, were analyzed to understand the structural and morphological alteration of MF to modified MF.

### 2.4. Characterization

#### 2.4.1. Morphological and structural characterization

XRD analyses of the synthesized powder silica particles were carried out using a X-ray diffractometer (Bruker brand D8 advance model) with CuKα radiation at 35 kW and 15 mA (1.541871 A°). Powder silica particles were scanned from 10° to 80° (scanning speed of 2°/min). The surface morphology analyses of the amine functionalized silica particles were characterized by using SEM (ZEISS LS-10). FTIR spectra were employed using an ATR module-FTIR (Bruker, USA) spectroscopy in the range of 400–4000 cm^−1^.

#### 2.4.2. QCM-D analysis

QCM-D technique was used to confirm the accumulation of CHI, PSS, and silica on each other using a Q-Sense QCM-D E1 device. The sensor used during the experiments had a piezoelectric quartz crystal with Au coating (AT-cut, 4.95 MHz ± 50 kHz). CHI, PSS, and silica solutions were adsorbed on the precleaned quartz sensor sequentially. Frequency/Dissipation changes depending on adsorption on the sensor were monitored simultaneously via the software as a function of time and the number of deposition bilayers. The empirical equation that gives the relationship between the frequency change and the mass of the coating adsorbed on the sensor is the Sauerbrey equation (Equation 1).


(1) $$\Delta m=\frac{-C_{f}\times \Delta F}{n},$$

where ΔF denotes the frequency change, the n is a harmonic, which is an odd number like 1, 3, 5, 7, 11, 13 and C is a constant showing the mass sensitivity of the 5 MHz crystal (17.7 ng cm^−2^ Hz) [21].

#### 2.4.3. Contact angle measurements

The wettability of the foams with water, organic solvents with different densities, and oil was monitored. At the same time, the effect of the modification on the WCA was examined. For this purpose, the static WCAs of the foam surfaces were determined by the sessile drop technique using the KSV Attension Tensiometer system.

### 2.5. Sorption capacity

The sorption capacities of the modified foams for selected model pollutants and water were statically measured. After the foam was weighed; it was immersed in the selected liquid and kept for a while until it became saturated. Subsequently, the dripping parts of the model pollutant from the foam surface were swept with tissue paper. Afterward, they were quickly weighed again, and the weight was recorded. The sorption capacity was calculated according to the following equation (Equation 2).


(2) $$q_{t}=\frac{w_{t}-w_{i}}{w_{i}},$$

where q_t_ is the model pollution sorption capacity of the foams, w_i_ and w_t_ are the weight of the foams before and later sorption, respectively. Each test was repeated at least five times.

### 2.6. Compressive mechanical test

Cyclic compression test with a strain of 50% was carried out to assess the mechanical performance of the modified and unmodified MFs with average dimensions at about 10.0 ± 0.7 mm, 9.8 ± 0.7, 9.8 ± 0.6 mm. Ten times cyclic compression test was performed on the foams at a strain rate of 10 mm min^−1^ at ambient temperature using a universal Shimadzu AG-IS mechanical tester equipped with a 50 N load cell. A preload of 0.1 N was applied to ensure complete contact before testing. Elastic modulus was determined for each cycle as the slope of the linear region of the stress-strain curve over a 1.5% strain range from 0% to 10%. The stress at 50% strain was described as compressive strength. The compression tests were repeated 3 times.

## 3. Results and discussion

### 3.1. Characterization of silica particles

**Figure 3:**
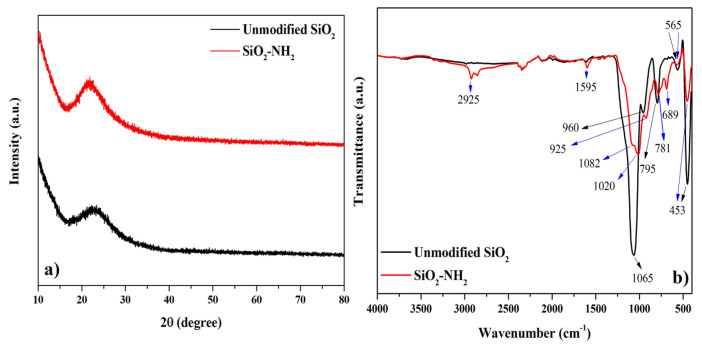
a) XRD and b) FTIR graphs of the silica particles.

XRD diffractograms of the unmodified SiO_2_ and amino-modified SiO_2_ particles (SiO_2_-NH_2_) are shown in Figure 3a. After modification, no sharp peak was observed for SiO_2_-NH_2_ particles which had the typical peaks for SiO_2,_ and a typical broad peak was observed at 2θ =15° to 30° for all samples (JCPDS card number: 29-0085) [22]. This implied that the main structure of the synthesized silica particles was amorphous [23,24] and its structure did not change after modification. It was observed that only peak intensities increased after modification with APTES. Also, no other impurity phases were observed. This finding confirmed the purity of the prepared silica particles.

FTIR analysis was used for confirming the modification of silica particles. Figure 3b displays the FTIR spectrum of the unmodified SiO_2_ and SiO_2_-NH_2_ particles. Blue and black arrows indicated the bands of SiO_2_-NH_2_ and unmodified SiO_2_ particles, respectively. In the FTIR spectrum of the unmodified SiO_2_, a band was seen at 960 cm^−1^ corresponding to Si-OH group (see in Figure 1). The FTIR bands at 453 and 795 cm^−1^ were also assigned to the asymmetric stretching of the Si-O-Si group [7,25]. Furthermore, the band around 1065 cm^−1^ was attributed to the asymmetric stretching of Si-O-Si which confirmed the silicon dioxide structure [25–27]. A weak band at 565 cm^−1^ was attributed to the characteristic band of Si-O [27]. Concerning the FTIR spectrum of SiO_2_-NH_2_, the characteristic peaks of SiO_2_ at 565 and 453 cm^−1^ appeared, but the peak intensities decreased. In addition, it was determined that the peaks at 960 and 795 cm^−1^ shifted to 925 and 781 cm^−1^, respectively. This can be due to the consumption of OH groups of the SiO_2_ surface during the modification process by APTES [28]. The peak in 1065 cm^−1^ appeared at 1020 cm^−1^ with a shoulder while its intensity declined. At the same time, the new bands at 2925, 1595, and 689 cm^−1^ were related to -CH_2_ absorption peak (-OC_2_H_5_ group of APTES), -NH- bending vibration of amine groups, and Si-CH_2_-, respectively [4,28]. Therefore, the presence of new functional groups and the changes in the characteristic peaks of SiO_2_ indicated that the SiO_2_ particle was successfully modified.

**Figure 4:**
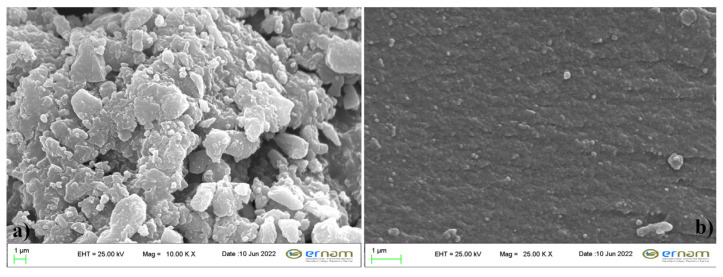
SEM photos of the amine functionalized silica particles.

The SEM photos of SiO_2_ particles are given in Figure 4 at different magnifications with ×10,000 and ×25,000 (1 μm index). Based on the SEM photos given in Figure 4a, it can be said that SiO_2_-NH_2_ particles do not change to large-size clusters after the modification while silica particle clusters of various shapes and sizes were formed. At the same time, the morphology of SiO_2_-NH_2_ particles mostly showed irregular micro-flake shapes with agglomeration. Comparing the SEM photos in Figures 4b and 4a, spherical particles can be identified when the magnification was increased. This may indicate that large-size clusters were formed by the agglomeration of small spherical SiO_2_ particles.

### 3.2. QCM-D analysis

**Figure 5:**
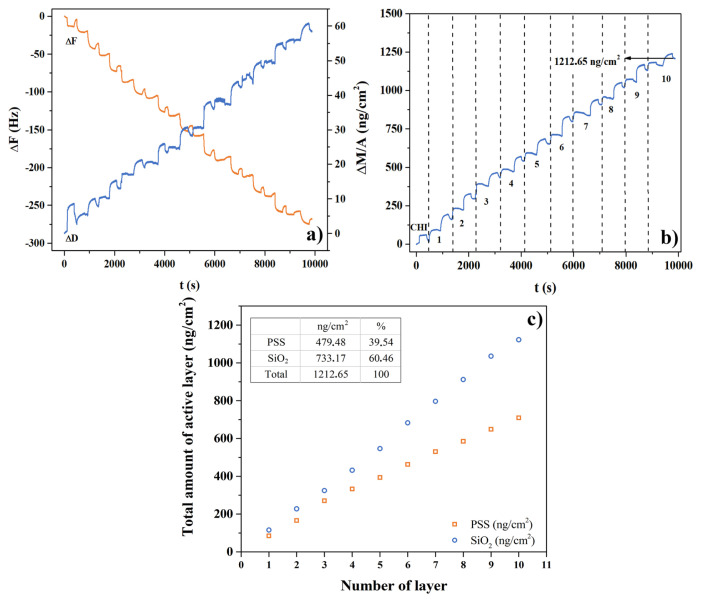
(a) Frequency (ΔF), dissipation (ΔD) changes, (b) change in mass (ΔM/cm^2^) of (CHI-PSS/SiO_2_)_10_ depending on time (n = 5), and (c) film composition of (CHI-PSS/SiO_2_)_10_ depending on the number of layers (n = 5).

The growth of CHI-PSS/SiO_2_ multilayers deposited on quartz crystal was monitored by a QCM-D system. In Figure 5a, the frequency shift versus time depending on sequential adsorption of CHI, PSS, and amine functionalized SiO_2_ layers are presented for LMF as an example. The first one was the chitosan layer leading positive charges on the foam surface, then the remaining bilayers were formed with PSS and amine functionalized SiO_2_. While the frequency of the quartz sensor decreased with the number of deposited bilayers, the dissipation value of the multilayered film was raised. The decline in the frequency value implied rising in the mass accumulated on the sensor according to the Sauerbrey equation. After ten-bilayer sequential adsorption, approximately 1212 ng film consisting of 60% SiO_2_ and 40% polyelectrolyte was deposited per unit sensor area (Figure 5c). The SiO_2_ was more than PSS in the film combination. It was related to balancing the negative charges occurring in the PSS structure, which was known as a high charge density polyelectrolyte. This assessment was also confirmed by the dissipation variation. The dissipation value of approximately 60 × 10^−6^ indicated a relatively soft and viscous multilayered film structure because SiO_2_ caused some holes and gaps in the polymeric structure [21,29].

### 3.3. Structural and morphological properties of the foams

**Figure 6:**
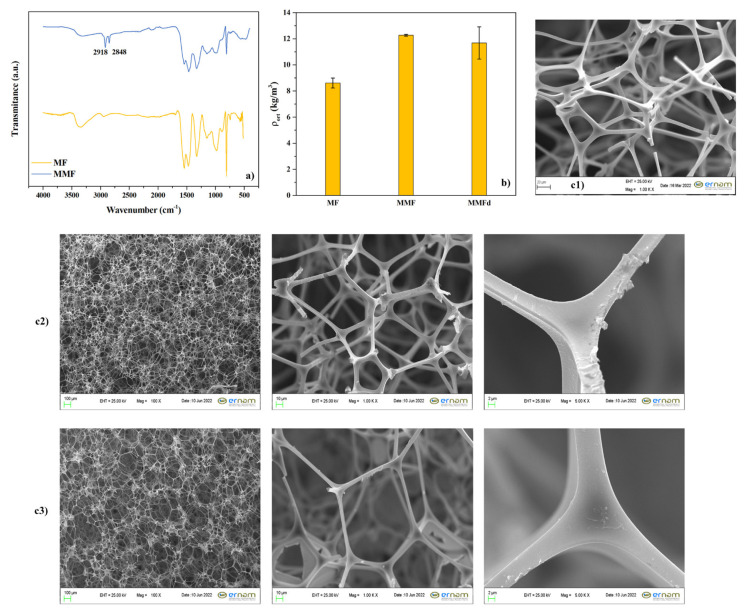
(a) FTIR spectrum, (b) foam density, and (c) SEM photos of unmodified and the modified foams (c1: MF, c2: MMF, c3: MMFd).

FTIR analysis was performed to determine the chemical structures of the modified and unmodified foams and their spectra are given in Figure 6a. The results confirmed the modification with hydrocarbon thiol compounds according to the new peak at about 2918 and 2848 cm^−1^ assigned to C-H and S-H stretching vibration, respectively [30,31]. In addition, the increase in the density of the foam calculated according to the ASTM D1622-03, (seen in Figure 6b) confirmed the presence/deposition of the modification agents on the cell walls of MF (ρ_MF_ = 8.61 ± 0.38, ρ_MMF_ = 12.27 ± 0.10, ρ_MMFd_ = 11.68 ± 1.23) [32]. The changes in MF’s morphology after modification using both the LbL-like approach and dip coating technique were examined with SEM analysis and the obtained photos are shown in Figures 6c1–6c3. The unmodified MF had an open cell structure, and it showed smooth surfaces on the column and cell wall (Figure 6c1). After modification with CHI, SiO_2_, and thiol compound, it appeared that the modification agents were deposited on the colon walls with the shape of a wrinkled layer. In connection with this, the colon wall thicknesses of the polymeric foam were found to be 6.86 (± 1.68), 8.62 (± 1.54), and 12.78 (± 2.62) for MF, MMF, and MMFd, respectively from SEM photos using ImageJ analysis program. It can be seen from Figures 6c2 and 6c3 that the MF kept its open cell structure. On the contrary, to the LbL-like approach, many pores were found to be clogged after the direct dipping method. It was thought that the washing steps of the modified foam prepared with the LbL-like approach ensured the prevention of clogging and it maintained the open pore structure.

### 3.4. WCA of the MF and modified foams

**Figure 7:**
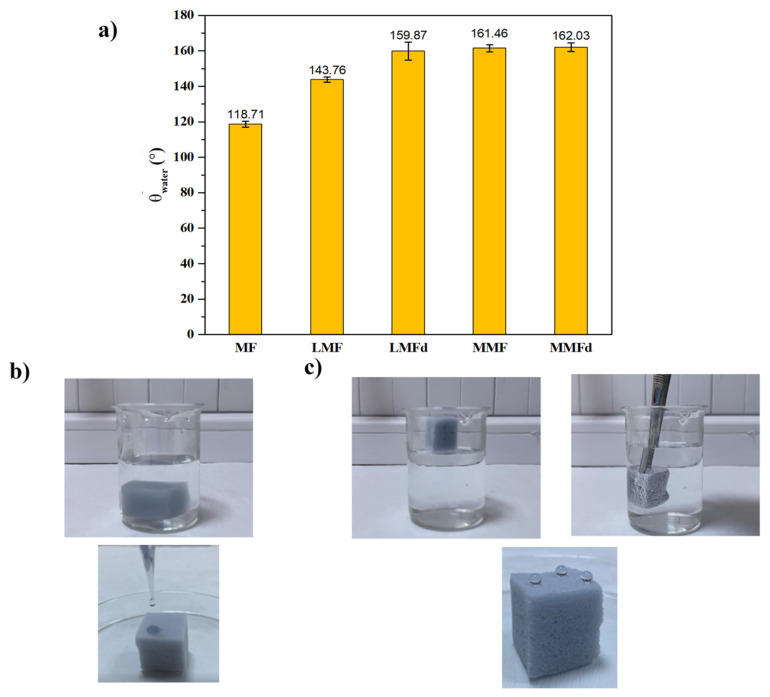
(a) WCAs of the foams, digital photos of water droplets on (b) MF and (c) MMF, and their behavior in water.

An important property that is as critical as structural properties such as porosity and surface roughness for sorbents used in oil sorption is wettability. The ability to remove an oily pollutant from the water surface or inside the water mass substantially depends on the sorbent repelling water as much as it attracts oil. Since hydrophobicity is one of the main features affecting the sorption capacity and selectivity of sorbents, the WCAs of the modified sponges were determined [33,34]. In Figure 7a, the WCA values of the foams are given before and later the modification. Also, the photos can be seen in Figures 7b and 7c. MF had a WCA of 118.71° (± 1.73) (Figure 7a). After silica coating with a LbL-like approach, this value increased to about 143.76° (± 1.50) (LMF), while it further rose to 159.87° (± 5.09) via direct dipping using CHI/SiO_2_ solution (LMFd). Both the Cassie–Baxter and Wenzel theories state that the surface roughness must be increased to alter the hydrophilic or hydrophobic character of a surface [13,35]. These results indicated that the cell morphology was changed, and a rougher surface was obtained compared to MF after silica modification according to the literature [6,17]. The evaluation of WCA was supported by the SEM analysis. Subsequently, a WCA of 162° was provided for the modified MF after the final modification with hydrocarbon thiol compound regardless of the used methods (MMF (161.46° ± 1.94) and MMFd (163.03° ± 2.46)). Long hydrocarbon chains of the thiol structure led to significant enhancement in the superhydrophobic behavior and water/oil selectivity of the modified foams.

Another attempt was made to understand the wetting behavior and affinity of MF and MMF to water. When MF was placed in a water-filled beaker, it sank immediately (Figure 7b). In contrast to MF, MMF remained on the water’s surface. Further, a silver mirror image was observed when MMF was immersed in the water by applying force (Figure 7c). In other words, MF, which can sorb both water and organic solvent/oil, lost its affinity to water after modification and did not sorb water. These results confirm that hydrophobic modification of MF was successfully performed.

### 3.5. Sorption performance and recyclability of the foams

**Figure 8:**
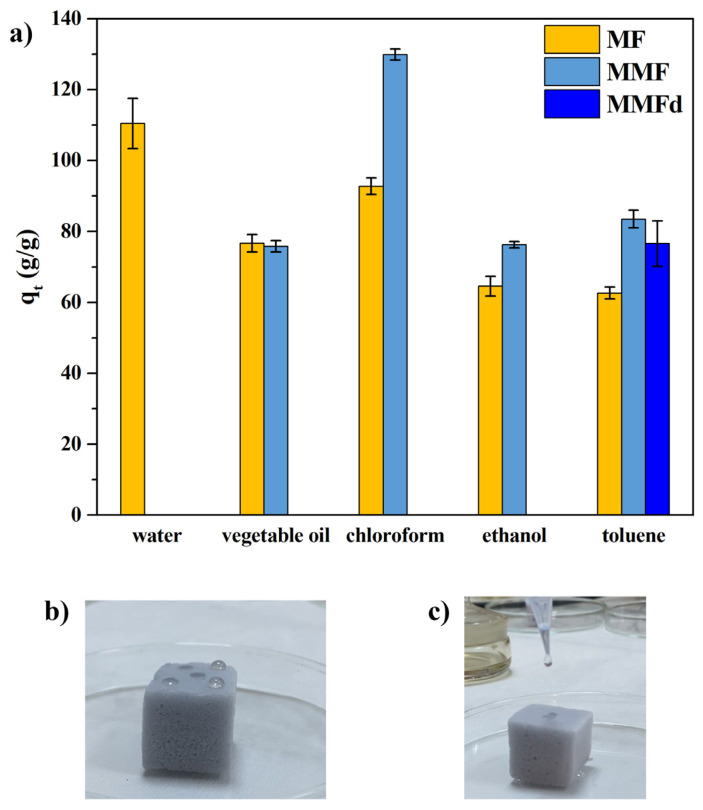
(a) Sorption capacity of the foams for different model pollutants, (b–c) digital photos of vegetable oil and toluene droplets on the MMFd surface, respectively.

Figure 8a shows the sorption capacity of MF, MMF, and MMFd for several model pollutants such as vegetable oil, chloroform, ethanol, and toluene. MMF sorption capacities were calculated according to Eq. 2 and they were found to be 76, 130, 76, and 84 g/g for vegetable oil, chloroform, ethanol, and toluene, respectively. These values were up to 40% higher than that of the sorption capacity of MF ranging from 62 g/g to 93 g/g for selected model impurities. The changes in sorption capacity could be explained by the difference in density of the pollutant because the sorption capacity of sorbents is heavily affected by the density and viscosity of pollutants such as oil and organic solvent [31]. Furthermore, the increase in sorption capacity of MMF with the use of the higher density of organic solvents as a pollutant (ρ_chloroform_ > ρ_toluene_ > ρ_ethanol_) indicated that the sorption capacity is directly connected with the density.

In contrast to the sample prepared with the LbL-like approach, it was observed that MMFd had a less homogenous structure than that of MMF, even though its toluene sorption capacity was higher than MF. As can be seen in Figures 8b and 8c, the vegetable oil droplet was partly sorbed and in some regions remained spherical form on the surface without sorption by MMFd while it sorbed the toluene droplet on its surface. This was an unexpected behavior considering the WCA of this sample (around 162°). However, SEM results showed that the pores in the MMFd structure were clogged in some regions. Therefore, this situation can be explained by the presence of SiO_2_/CHI mixture, which accumulated in the pores and restricted the movement of the model pollutants between the channels through absorption. For the MMF, it was evaluated that the washing step in the preparation method prevented this situation.

**Figure 9:**
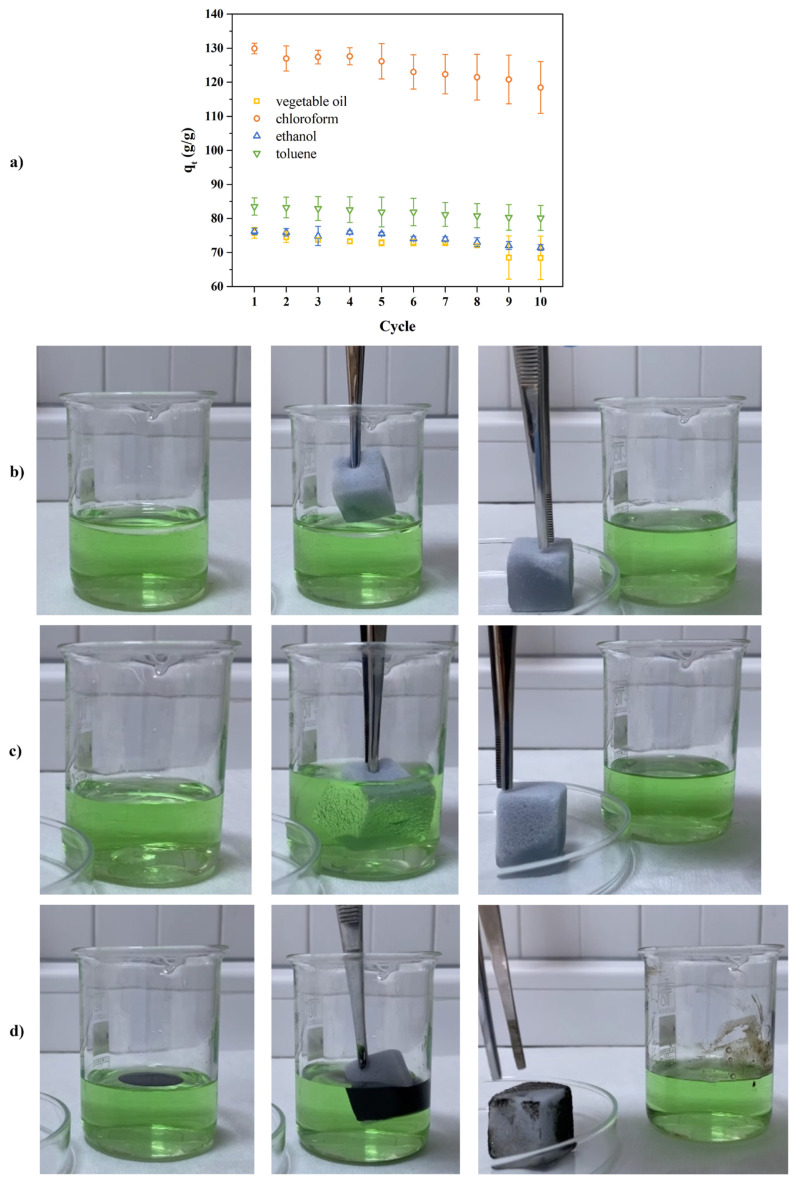
(a) Sorption capacity of MMF against several model pollutants up to 10 cycle, (b–d) photos of toluene sorption on water surface, chloroform sorption at the bottom of water and waste motor oil sorption on water surface with MMF, respectively (water is colored with green food dye).

The recyclability of MMF was examined by the repetitious cycle of the sorption and release process and the sorption capacities obtained at each step for the different pollutants are presented in Figure 9a. It was found that MMF maintained its flexibility while its sorption capacity remained almost the same after 10 cycles. After 10 sorption/release cycles, the sorption efficiency decreased by 9.7%, 8.8%, 6.3%, and 4.0% for vegetable oil, chloroform, ethanol, and toluene, respectively. It was observed that this decrement was higher for a more viscous and dense pollutants. This reduction may be explained by the shrank of the foam cell in the squeeze step of the sorption/release cycle, and may also be due to the presence of pollutant between the layers throughout the LbL structure [36].

To understand the behavior of MMF in practical applications, its affinity for toluene, chloroform, and waste motor oil in a water-filled beaker was investigated and the separation process is shown in Figures 9b–9d. It was observed that the foam sorbed model pollutants in a short time and did not soak in the water, which was colored green. The oil sorption mechanism can occur through absorption, adsorption, or both mechanisms (especially in biopolymeric materials). If the oil sorption mechanism happens by absorption, the pollution penetrates the pore spaces. At this point, the porosity of the sorbent and the properties of the pollutant such as viscosity, density, and adhesion come to the fore. Here, it is thought that selected pollutants with lower density and viscosity adhered to the modified MFs predominantly through the absorption mechanism (Figures 9b and 9c). This is why MMFd which has some clogged pores had a lower sorption capacity than MMF. In the literature, it has been reported that the mechanism works through adsorption when the pollutants are linked to the sorbent surface by various chemical and physical interactions. In this case, it meant that the adsorption mechanism was prominent in the sorption of waste motor oil (Figure 9d). In particular, it is assumed that the LbL-based sorbents with increased functionality took place through hydrophobic interactions and Van der Waals forces [37,38].

### 3.6. Mechanical behavior of the foams

**Figure 10:**
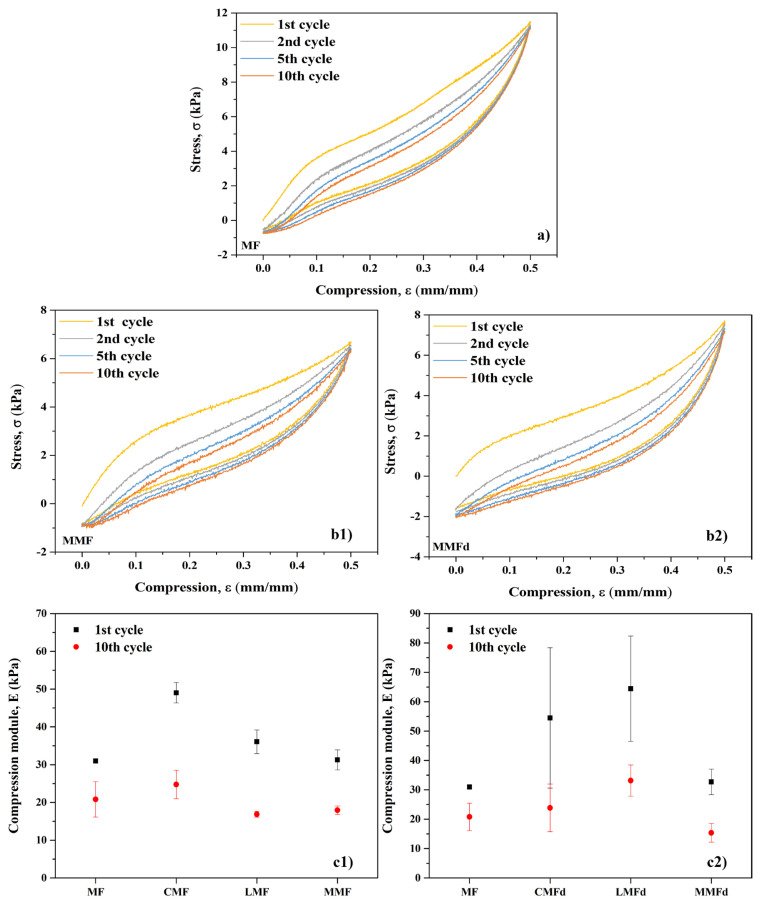
(a) Stress versus strain curve of (a) MF and (b1–b2) MMF and MMFd, prepared by LbL and dip coating methods, respectively; (c1-c2) compressive modulus of unmodified and the modified foams at a strain of 50% and a strain rate of 10 mm min^−1^.

The cyclic compression curves of MF, MMF, and MMFd are shown in Figures 10a and 10b. Hysteresis loops were found in the stress-strain curves of all tested foams during the loading and unloading process. This indicated the energy dissipation properties of the tested foams, which would enable the foam to achieve a well-cushioning function [39]. The compressive strength of MF was found to be 11 kPa at 50% strain, which is well suited to the literature. The stress-strain curve had an approximate linear elastic regime at the initial strains below 10% because of the elastic bending of the foams as reported by Shen et al. [40]. The compressive modulus derived from the slope of the linear area ranged from 15.37 ± 3.15 to 62.11 ± 9.07 (Figure 10c). The compressive modulus of the foams is inclined to decrease by 33%–56% with the number of cycles at a constant strain of 50%. CHI/SiO_2_ introduction increased in the compressive modulus of the foams. The main reason is that CHI/SiO_2_ injection to MF leads to a denser structure (Figure 6b) which may enhance the compressive strength [41]. However, a further thiol modification to CHI/SiO_2_ caused a slight decrease in the mechanical performance. MF modification did not affect the compressive modulus of the foams and they remained at the same level regardless of modification routes. Moreover, the compression strength and compressive modulus of MF, MMF, and MMFd foams remained at high levels, indicating the constant mechanical stability of the foam structure after 10 times cyclic compression [40,42]. For instance, the compressive strength of MMF and MMFd foams dropped by only 5% (from 6.65 to 6.31 kPa and from 7.67 to 7.14 kPa, respectively).

## 4. Conclusion

In summary, we prepared flexible, mechanically stable, and superhydrophobic modified foam sorbent for oil/organic solvent-water separation using the commercial MFs as a framework. It was determined that the use of the LbL-like approach as a modification technique allowed the preparation of more stable and homogeneous sorbents. The modified MMF had a high sorption capacity compared to MMFd and unmodified MF, which could sorb several model pollutants ranging from 76 g/g to 130 g/g depending on the pollution density and type. Up to 10 cycles of sorption/release process, MMF with a high-WCA (approximately 162°) remained nearly the same in both its mechanical stability and sorption capacity. MMF is thought to be a promising material in practical water-oil separation processes because of its easy preparation from relatively cheap materials and its advantageous properties.

## Figures and Tables

**Table:** Sample codes for the MF and the modified foams according to their preparation routes.

Sample codes	Definition of sample codes
MF	Unmodified melamine foam
CMF	CHI coated melamine foam via LbL-like approach (with washing step)
CMFd	CHI solution impregnated melamine foam via dip coating technique (without washing step)
LMF	CHI/PSS/amine functionalized SiO_2_ coated melamine foam via LbL-like approach
LMFd	CHI/SiO_2_ solution impregnated melamine foam via dip coating technique
MMF	1-octadecanethiol modified LMF
MMFd	1-octadecanethiol modified LMFd
